# Deciphering evolutionary dynamics of *SWEET* genes in diverse plant lineages

**DOI:** 10.1038/s41598-018-31589-x

**Published:** 2018-09-07

**Authors:** Xiaoyu Li, Weina Si, QianQian Qin, Hao Wu, Haiyang Jiang

**Affiliations:** National Engineering Laboratory of Crop Stress Resistance, School of Life Science, Anhui Agricultural University, Hefei, 230036 China

## Abstract

*SWEET*/*MtN3*/*saliva* genes are prevalent in cellular organisms and play diverse roles in plants. These genes are widely considered as evolutionarily conserved genes, which is inconsistent with their extensive expansion and functional diversity. In this study, *SWEET* genes were identified from 31 representative plant species, and exhibited remarkable expansion and diversification ranging from aquatic to land plants. Duplication detection indicated that the sharp increase in the number of *SWEET* genes in higher plants was largely due to tandem and segmental duplication, under purifying selection. In addition, phylogeny reconstruction of *SWEET* genes was performed using the maximum-likelihood (ML) method; the genes were grouped into four clades, and further classified into 10 monocot and 11 dicot subfamilies. Furthermore, selection pressure of *SWEET* genes in different subfamilies was investigated via different strategies (classical and Bayesian maximum likelihood (Datamonkey/PAML)). The average dN/dS for each group were lower than one, indicating purifying selection. Individual positive selection sites were detected within 4 of the 21 sub-families by both two methods, including two monocot subfamilies in Clade III, harboring five rice *SWEET* homologs characterized to confer resistance to rice bacterial blight disease. Finally, we traced evolutionary fate of *SWEET* genes in clade III for functional characterization in future.

## Introduction

The Sugars Will Eventually Be Exported Transporters (*SWEET*) gene family, is ubiquitous in plants, and plays diverse physiological and biological roles^1–7^. The first gene of *SWEET* family was identified as *MtN3* in *Medicago truncatula*, which is involved in the Rhizobium-induced nodule development^1^. Later, a homolog of the *MtN3* gene was found in Drosophila. This homolog is expressed in embryonic salivary glands and was named *saliva*; thus, this type of gene was initially described as a *MtN3*/*saliva* gene. Most *SWEET* genes encode proteins that harbour two MtN3/saliva (MtN3_slv) domains, that consist of 3 + 1 + 3 transmembrane helices. Only a few encode proteins that harbor 3 transmembrane helices that constitute one MtN3_slv domain^7,8^. Subsequently, members of the *MtN3*/*saliva* gene family have been predicted or characterized to be involved in various physiological processes in plants^1–7^. One of the most fascinating discoveries was that these genes can transport sucrose across the plasma membrane, and this family was finally named the *SWEET* gene family^7,9^.

Sucrose, which is the predominant type of fixed carbon transported in plants^9,10^, is synthesized in mesophyll cells, imported into phloem cells and subsequently transported to heterotrophic “sinks” (meristems, roots, flowers, and seeds). In this “phloem loading” process, sucrose is first effluxed from phloem parenchyma cells by SWEETs and then loaded into the sieve element-companion cell complex (SE/CC) via active proton-coupled sucrose transporters (SUTs)^11,12^. Sucrose translocation has critical importance in basic physiological processes such as reproductive development, senescence, and in the allocation efficiency of plants, which is closely associated with crop yield^6,13^. In a more recent report, *ZmSWEET4c* in maize and its rice ortholog *OsSWEET4*, which mediates hexose transportation, were shown to influence seed filling and size^14^. Furthermore, the *SWEET* genes involved in this sugar efflux system have been shown to be hijacked by pathogens^5,15,16^. At least three *SWEET* genes are involved in the resistance to various *Xanthomonas oryzae* pathovar *oryzae* (Xoo) strains, which cause one of the most devastating global rice diseases. The dominant alleles of the recessive resistant gene *OsSWEET11* (*xa13*), *OsSWEET13* (*xa25*) and *OsSWEET14* (*xa41*), are induced by the various Xoo strains in the promoter region, suggesting that they supply sugar to pathogens^5,16,17^. In a susceptible reaction, their promoter regions are specifically targeted by bacterial type III effector genes produce four different type TAL (transcriptional activator-like) effectors. Furthermore, another two rice *SWEET* genes that are phylogenetically close to the three rice *SWEET* resistance genes have also been inferred to be Xoo virulence targets, and may be *R*-genes^18^.

The functional importance of many *SWEET* genes as ubiquitous transporters remains elusive. No comprehensive survey has been conducted in *SWEET* genes in plant taxa. To date, most investigations on *SWEET* genes have focused on a few species at the whole-genome scale, including *Arabidopsis thaliana*, rice, soybean, and tomato^7,8,19–21^. With limited data and results, *SWEET* genes are believed to have been extensively conserved, but this not agree with its observed functional diversity and continuous expansion and duplication^8^. In this study, *SWEET* genes were characterized in 31 plant genomes, ranging from single-celled plants to higher terrestrial plants. The distribution and duplication models of SWEET genes were also explored here. Phylogenetic reconstruction and molecular evolution analyses of *SWEET* genes revealed their evolutionary genetic basis.

## Results

### Genome-wide identification of *SWEET* genes in 30 representative plant species

**Figure 1:**
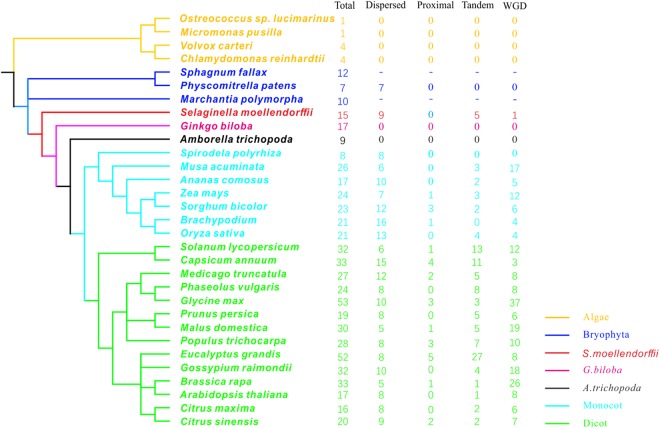
Species tree of 31 plant species and duplication modes estimation of *SWEET* genes. Species from different taxonomy or species were marked with different colour; −means duplication mode could not be estimated.

In this study, *SWEET* genes were systematically surveyed in 31 plant genomes, ranging from aquatic algae to angiosperms. A total of 636 *SWEET* homologs were identified among our sampled genomes (Fig. 1 and Supplementary Table S1). Interestingly, *SWEET* genes were detected in unicellular aquatic algae, which was indicative of its ancient origin and functional conservation. In addition, the numbers of *SWEET* members in land plants indicated varying degrees of expansion compared to aquatic algae. Firstly, only one, one, four and four homologs were identified in four aquatic algae *O*. *lucimarinus*, *M*. *pusilla*, *C*. *reinhardtii* and *V*. *carteri*, respectively, all of which were remarkably fewer than those found in land plants. Secondly, in the lower land plant *P*. *patens*, which is believed to be one of the earliest land lineages that diverged from aquatic plants^22^, and *A*. *trichopoda*, which is the single living representative of the sister lineage of all living angiosperms^23^, seven and nine *SWEET* genes were identified, respectively. Whereas, in the non-seed lycophyte *S*. *moellendorffii*, 15 *SWEET* homologs were found. In *G*. *biloba*, a gymnosperm species that is described as a living fossil, 17 *SWEET* homologs were characterized. In the seven monocot species, eight to 26 *SWEET* genes were identified. In eudicots, 16 to 53 *SWEET* genes were observed, suggesting extensive gene expansion and duplication events. Most of the *SWEET* genes were observed in the legume plant, *G*. *max* and the rosids plant *E*. *grandis*, which harbored 53 and 52 *SWEET* homologs, respectively. Although copy number variations among species were apparently complex, our data suggested that the number of *SWEET* homologs in each species was positively correlated with genome-wide gene numbers (r = 0.7168, *P*-value = 3.79e-05) (Supplementary Fig. S1). In addition, the distribution of *SWEET* homologs was not evenly distributed within one species or among plant lineages. For example, no *SWEET* genes were observed in four of the 12 rice chromosomes, whereas, roughly 57.1% of the rice homologs were detected on chromosomes 1 and chromosome 9. In the two legume genomes *G*. *max* and *M*. *truncatula*, both the copy number and distribution of *SWEET* homologs were distinct (Supplementary Fig. S2).

Furthermore, the characterized SWEET proteins from various species generally fell into two types. Most of these proteins harbor two MtN3_slv domains, whereas a few consist of one MtN3_slv domain^7,8^. Herein, a comprehensive investigation of the number of MtN3_slv domains was conducted on all 31 plants (Supplementary Fig. S3 and Table S1), and 90% of the predicted SWEET proteins contained two MtN3_slv domains, including all homologs from three unicellular plants. SWEET proteins that only harbored one MtN3_slv domain were observed in *P*. *patens*, as well as in most multicellular plants except for *S*. *bicolor*, *A*. *thaliana*, *P*. *vulgaris* and *C*. *grandis*. Interestingly, one *SWEET* homologs, which were characterized in *E*. *grandis*, consisted of three MtN3_slv domains.

### Expansion models of *SWEET* genes among plant genomes

Gene expansion or duplication, which frequently occur in plant taxa, is often followed by divergence, thereby resulting in subfunctionalization, novel evolutionary materials and adaptive advantages^24,25^. Diverse duplication models such as whole-genome duplication (WGD) or segmental duplications (SD), local duplication (including tandem and proximal duplications) and dispersed duplication), have been hypothesized for gene duplication^24–27^. Each of these models is biased in regard to gene retention by either contributing to genetic redundancy or evolutionary novelty^26^. Hence, estimation of the duplication model of *SWEET* genes was performed for the surveyed genomes via MCscanX software, including two multicellular algae, the basal land species *P*. *patens*, *S*. *moellendorffii* and all angiosperms (those species were excluded due to either having a of sing-copy SWEET genes or poorly assembled genomes) (Fig. 1)^28^. The results revealed that the proportions of *SWEET* genes retained from different gene duplication models differed within or among species. Interestingly, dispersed duplication was the only duplication mode detected within all of the surveyed species. Furthermore, dispersed duplication was also the only duplication mode in *SWEET* genes from two algaes and *P*. *patens*. WGD/segmental duplication events involving *SWEET* genes were observed in each higher plant species, but not in mosses and algae, which may be related to the phenomenon that all vascular plants undergo one or more whole-genome duplication events. At least three types of duplication events in *SWEET* genes were detected in every surveyed angiosperm except for the aquatic moncot *S*. *polyrhiza*. In particular, *SWEET* genes retained from dispersed, proximal, tandem, and WGD/segmental duplication accounted for 37.2%, 4.6%, 19.4%, and 38.7% of the duplication events, respectively. The sharp increase in the number of *SWEET* genes in higher plants was largely due to segmental and tandem duplication compared with basal land plants. The proportion of these two types of duplication models in each species was not equal, and a species-specific duplication model preponderance was detected. For example, in monocots, WGD/segmental duplication was preferentially enriched in *M*. *acuminata* and *Z*. *mays* to a greater degree than in all of the other surveyed monocot plants. Conversely, tandem duplication mainly contributed to the expansion of *SWEET* genes in the two *Solanaceae* plants. For the only two species harboring more than 50 *SWEET* genes, 69.8% of genes in *G*. *max* were derived from WGD/segmental duplication events (Supplementary Fig. S3), while 52.0% of genes in *E*. *grandis* were derived from tandem duplication, which were much higher than those in the other species.

### Evolutionary rate estimation of duplicated *SWEET* paralog genes

Considering the important role of WGD/segmental duplication and segmental duplication in *SWEET* gene expansion, an estimation of the evolutionary dynamics of *SWEET* duplicated pairs would help to understand their evolutionary process in all surveyed angiosperms including dicot and monocot lineages. The dN/dS ratio is an important parameter for estimating molecular evolutionary rates and reflects the dynamics that drive evolution. Generally, a dN/ds ratio larger than 1 indicates positive selection and a dN/dS ratio less than 1 suggests purifying selection. In the present study, the dN/dS values of most duplicated paralogous genes were lower than 1 except for three gene pairs, which strongly indicated that most of these duplicated pairs experienced purifying selection. The three gene pairs, *Eucgr*. *F02750*/*Eucgr*. *F02751*, *Gorai*. *001G055600*/*Gorai*. *001G055700*, and *Glyma*. *05G036500*/*Glyma*. *17G090800*, exhibited dN/dS values larger than 1, suggesting that they underwent positive selection pressure during their evolutionary history.

**Figure 2:**
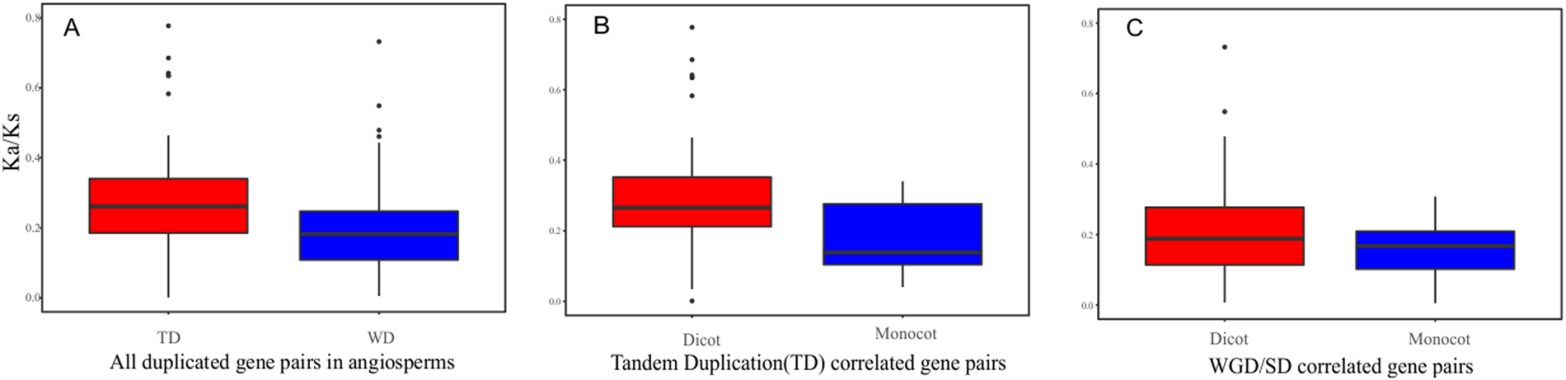
Ka/Ks values of SWEET genes in angiosperm plants. (**A**) Ka/Ks values of WGD/SD and tandem duplication genes pairs in plants. (**B**) Ka/Ks values of WGD/SD duplication gene pairs in dicot and monocot plants, respectively. (**C**) Tandem duplication gene pairs in dicot and monocot plants, respectively.

Furthermore, these results show the different evolutionary rates of WGD and TD duplicated pairs in angiosperms (Fig. 2). Comparing all of the WGD and TD duplicated pairs in angiosperms, the average dN/dS value of WGD (0.25) was less than that of the TD duplicated pairs (0.32). Comparing these two types of duplicated pairs in only monocot or dicot lineages, the average dN/dS value of WGD was less than that of the TD duplicated pairs. Smaller dN/dS values indicated WGD gene pairs evolved more slowly. Finally, both WGD and TD pairs in dicots had a higher average dN/dS value than that in monocots, reflecting the difference between the evolutionary rates of monocot and dicot duplicated SWEET pairs.

### Phylogenetic analysis of *SWEET* genes in 30 plant species

**Figure 3:**
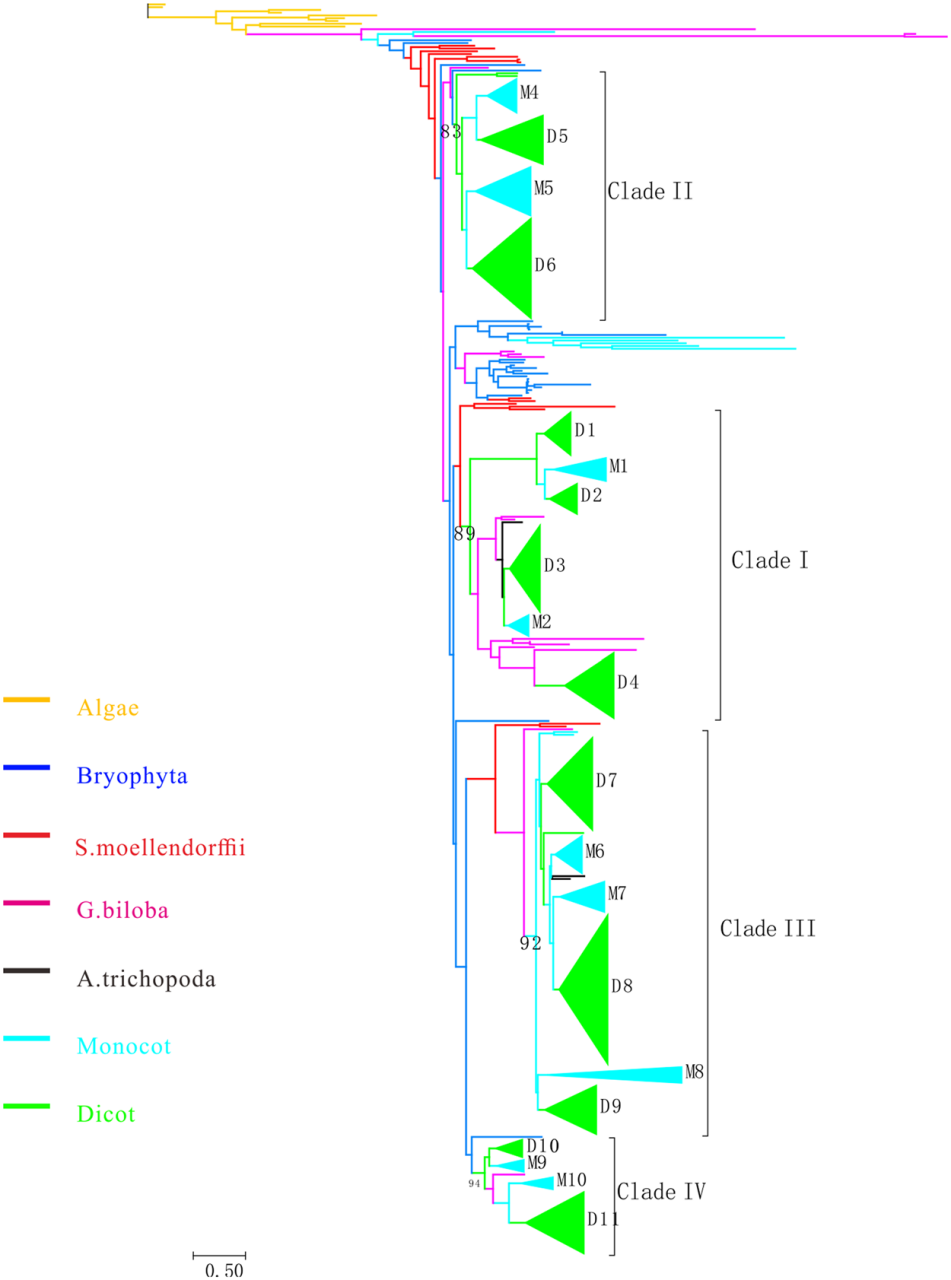
Maximum-likelihood (ML) phylogenetic tree built by *SWEET* genes from 31 plant species. Trees were built with the reliability of internal nodes and evaluated using the Shimodaira-Hasegawa approximate likelihood ratio test (SH-aLRT) values in PhyML 3.1 and were further edited by MEGA 5.0. The phylogenetic tree had exactly the same topological structure described by Chen Li *et al*.^7^ and could be divided into four clades, the major nodes of which were supported with high confidence (≥0.80). We followed the nomenclature of Chen *et al*. according to the distributing of the *SWEET* members in *A*. *thaliana*, and they are named clades I, II, III, and IV. Dicot and monocot SWEET clades were compressed to triangle.

To better explore the evolutionary history of *SWEET* genes in plants, complete protein sequences of SWEET genes were used to build ML trees (Figs 3 and S4). Our phylogenetic tree exhibited exactly the same topological structure described by Chen *et al*.^7^ was observed (Fig. 3 and Table 1). Thus, SWEET genes of angiosperm plants in the phylogenetic trees were also divided into four clades, and SWEET members in algae and basal land species, including three bryophyta plants, *S*. *moellendorffi* and *A*. *trichopoda*, were used as outgroups. Moreover, *SWEET* genes from *A*. *thaliana* were distributed among the four clades of the two phylogenetic trees, which was also consistent with the findings of the previous study^7^. We followed the nomenclature of Chen *et al*.^7^ and named these clades as I, II, III, and IV, in which 146, 120, 205, and 55 genes were characterized, respectively. Few large recently-duplicated subclades (gene number >5) were observed in the phylogenetic tree, except for three sub-clades in *E*. *grandis* (6, 6 and 13 genes, respectively) and one subclade in *M*. *domestica* (7 genes). These results indicated that a few extensive gene expansion events involving *SWEET* genes occurred in a species-specific manner; conversely, most expansion events took place before the taxonomic families or more ancient species diverged.

**Table 1:** Distribution of *SWEET* genes within four clades and 21 gene families.

Species	Clade I								Clade II					Clade III							Clade IV				
	Total	D1	D2	D3	D4	M1	M2	M3	Total	D5	D6	M4	M5	Total	D7	D8	D9	M6	M7	M8	Total	D10	D11	M9	M10
Musac	4	—	—	—	—	2	2	—	8	—	—	5	3	11	—	—	—	4	5	2	4	—	—	2	2
Ancom	2	—	—	—	—	1	1	—	6	—	—	2	4	5	—	—	—	4		1	2	—	—	1	1
Sppol	2	—	—	—	—	1	1	—	3	—	—	1	2		—	—	—	—	—	—	—	—	—	—	—
Zemay	5	—	—	—	—	1	2	2	5	—	—	3	2	11	—	—	—	5	4	2	3	—	—	1	2
Sobic	6	—	—	—	—	2	2	2	5	—	—	3	2	7	—	—	—	4	3	—	2	—	—	1	1
Bradi	5	—	—	—	—	2	2	1	4	—	—	2	2	6	—	—	—	2	3	1	2	—	—	1	1
Os	7	—	—	—	—	3	2	2	8	—	—	1	7	5	—	—	—	2	2	1	—	—	—	—	—
Capana	8		2	5	1	—	—	—	4	2	2	—	—	14	10	2	2	—	—	—	2	1	1	—	—
Solyc	10	1	2	6	1	—	—	—	5	3	2	—	—	13	10	2	1	—	—	—	2	1	1	—	—
Potri	11	3	1	4	3	—	—	—	3	1	2	—	—	8	4	3	1	—	—	—	6	2	4	—	—
Prper	6	1	1	2	2	—	—	—	5	1	4	—	—	6	1	2	3	—	—	—	2	—	2	—	—
Madom	13	2	6	4	1	—	—	—	7	2	5	—	—	9	2	4	3	—	—	—	1	—	1	—	—
Medtr	7	2	—	2	3	—	—	—	7	2	5	—	—	10	2	6	2	—	—	—	1	—	1	—	—
Phvul	6	2	—	2	2	—	—	—	7	2	5	—	—	10	3	6	1	—	—	—	1	—	1	—	—
Glyma	13	4	—	4	5	—	—	—	8	3	5	—	—	23	6	12	5	—	—	—	9	1	8	—	—
Eucgr	16	2	1	12	1	—	—	—	7	1	6	—	—	24	4	19	1	—	—	—	1	1	1	—	—
Gorai	7	1	2	2	2	—	—	—	6	3	3	—	—	11	4	5	2	—	—	—	7	2	5	—	—
Cigra	4	1	1	1	1	—	—	—	5	1	4	—	—	5	1	2	2	—	—	—	1	1	1	—	—
Cisin	4	1	1	1	1	—	—	—	6	1	5	—	—	5	1	2	2	—	—	—	4	1	3	—	—
Brara	7	3	—	2	2	—	—	—	7	2	5	—	—	15	2	12	1	—	—	—	3	—	3	—	—
AT	3	1	—	1	1	—	—	—	4	2	2	—	—	7	1	5	1	—	—	—	2	—	2	—	—

—Represents the absence of *SWEET* members in corresponding species within a clade or family; D, Dicot gene families; M; monocot gene families.

Interestingly, all the algal SWEET members clustered in one cluster and was apparently an outgroup, exhibiting co-orthologous relationship of all other plant *SWEET* genes (Figs 3 and S4). Whereas, *SWEET* genes in Clade II have relatively close relationship with the algal SWEET clade. Besides, each clade has nearby nested outgroups, constituted by SWEET members from all the surveyed basal land taxonomy (bryophyta plants and *S*. *moellendorffi*), indicating these four clades split as early as land plant speciation. The *SWEET* genes of the gymnospermous plant were also detected within all four clades. Additionally, all angiosperm plants could be found in every clade, except the aquatic moncot, *S*. *polyrhiza*. SWEET members in *S*. *polyrhiza* were absent in Clade III and IV. Finally, compared with the other three clades, clade III has the highest number of genes (205). Five rice *SWEET* genes in clade III have been reported to confer susceptibility to *Xoo*^18^, and may cause bacterial blight disease in rice. In the clade IV, the lowest number of genes (55) was observed.

### Molecular evolutionary analysis of *SWEET* genes

**Figure 4:**
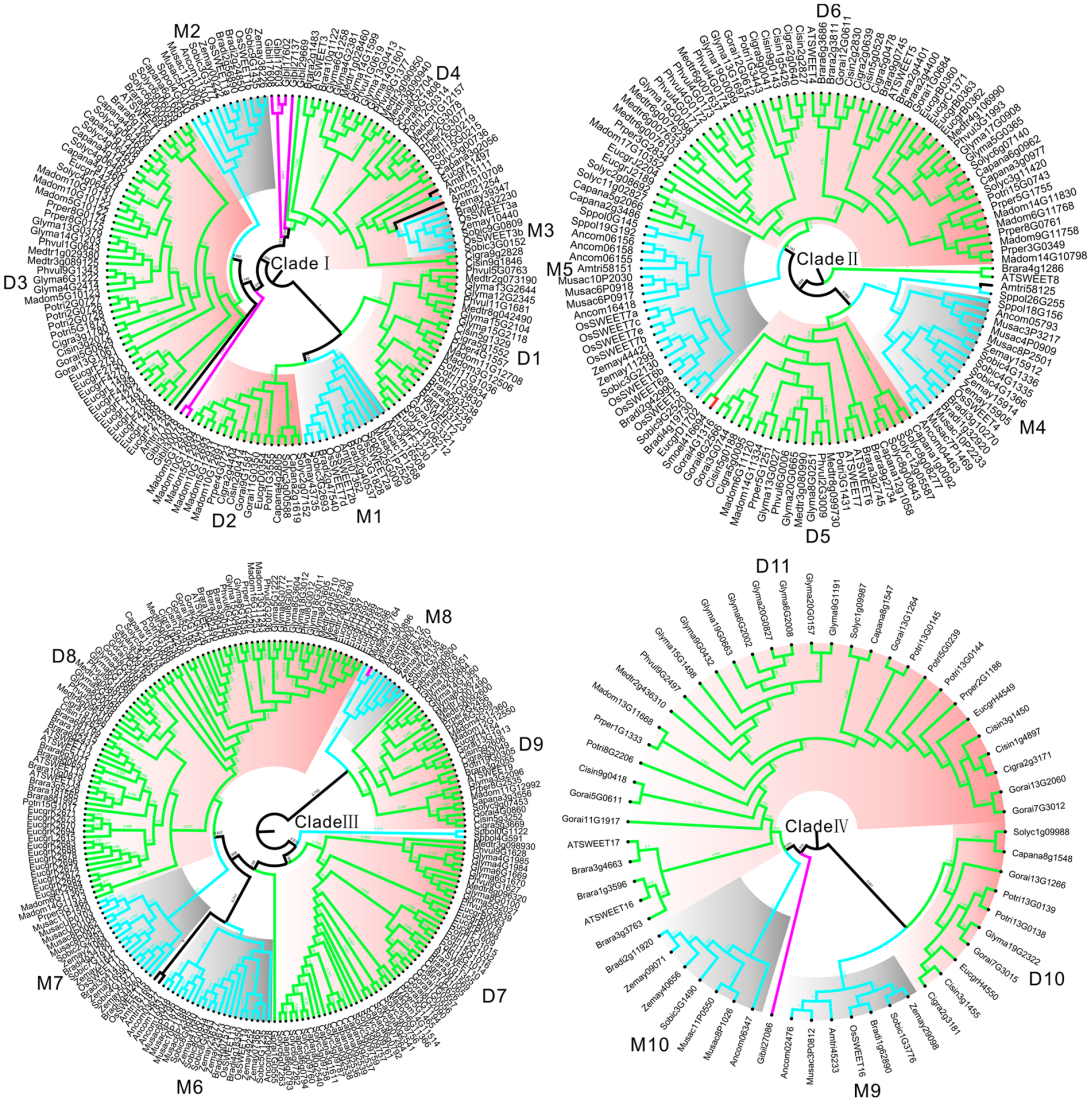
Subfamilies within different clades. Grey represents monocot-specific (M) subfamilies, and pink represents dicot-specific (D) subfamilies.

To better estimate the evolutionary rates of the expanded *SWEET* family in angiosperms, especially in dicots and monocot lineages, four clades in the phylogenetic tree were classified into distinct gene families. First, the monocot-specific (M) and dicot-specific (D) gene families were defined based on the following criteria: (1) According to the species tree (Fig. 1) and the distribution of homologs in *A*. *thaliana*, the M or D gene families should consist of homologs from most monocot or dicots species (not less than half of the dicots or monocots), (2) the clades in which the M or D gene families resided should have support values for basal nodes ≥0.70 (Fig. 4 and Table 1). These *SWEET* gene families were preserved throughout the evolutionary history of angiosperms and are regarded as a reliable core set of *SWEET* genes in angiosperms. Finally, 11 D gene families and 10 M gene families were explored, and these families accounted for the majority of all *SWEET* homologs. In the four clades we defined above, different numbers of M and D gene family members were characterized in each clade. Three M and four D in clade I, two M and two D in clade II, three M and three D in clade III, and two M and two D family were identified, respectively.

**Figure 5:**
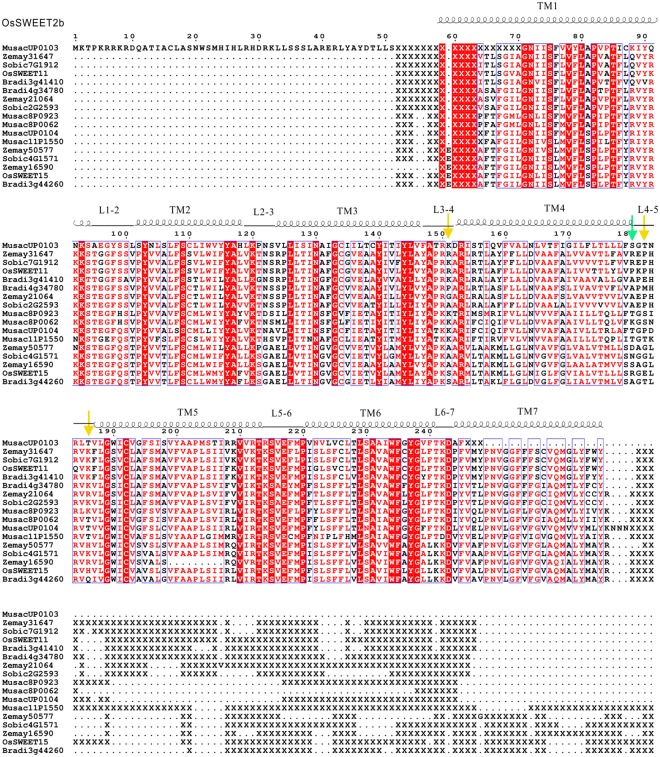
Sequence alignments of SWEET proteins in M7 and OsSWEET2b. The structure of OsSWEET2b was used as a reference to have the secondary structure assignment of SWEETs in M7. Positive selected sites are marked with arrows. Positive selection sites detected only by PAML are marked with yellow arrows; positive selection sites detected by both the two methods are marked with green arrows.

Firstly, possible recombination events, which may play important roles in differentiation, were also determined (See in Methods). Collectively, a total of 30 breakpoints were detected, and 19 (63.33%) occurred in nine M gene families, indicating that monocot *SWEET* families have a high recombination rate. Additionally, the two programs, namely, MEGA5.0 and PAML, were used to calculate the average ratio of non-synonymous to synonymous (dN/dS) for the M and D gene families (Table 2). The REL method in Datamonkey and branch-site approach in PAML were applied to detect individual sites under positive selection among the subfamilies. Positive selection sites were identified in 10 out of 21 subfamilies by at least one method. Whereas, positive selection sites were only identified in five subfamilies by both methods, including M2, M3, M7 and M8. Intriguingly, M7 and M8 belonged to clade III, and harbored the most genes. To better understand how positive selection was associated with gene function, we pinpointed the sites under positive selection of M7, that harbored one positive selection sites as identified by Datamonkey and four positive selection sites as identified by PAML. According to our data, three positive sites were detected by both methods. The sequences of M6 were aligned with MEGA and analyzed with the structure of *OsSWEET2b* (*Os01g0700100*, PDB number: 5CTG) as a reference^29^ (Fig. 5. We found that one positive selection site were located at the L2-3 region and three were at L4-5 (Fig. 5). The potential impact of these amino acid alterations on protein structure and function remain to be clarified.

**Table 2:** Estimation of the evolutionary parameters in CDS of *SWEET* genes in monocot-specific (M) and dicot-specific (D) families.

Subfamilies	Breakpoint	average dN/dS		Positive selected sites	
		PAML	MEGA	PAML	REL
D1 (n = 24)	2	kappa (ts/tv) = 2.22573	0.27	0	1
		omega (dN/dS) = 0.17350			
D2 (n = 17)	1	kappa (ts/tv) = 2.34758	0.27	3	0
		omega (dN/dS) = 0.20566			
D3 (n = 48)	0	kappa (ts/tv) = 2.09627	0.22	0	0
		omega (dN/dS) = 0.17602			
D4 (n = 26)	0	kappa (ts/tv) = 1.94345	0.24	0	0
		omega (dN/dS) = 0.20298			
D5 (n = 27)	1	kappa (ts/tv) = 1.76817	0.25	0	0
		omega (dN/dS) = 0.23493			
D6 (n = 55)	1	kappa (ts/tv) = 2.12555	0.28	0	0
		omega (dN/dS) = 0.25548			
D7 (n = 51)	2	kappa (ts/tv) = 1.77940	0.26	0	0
		omega (dN/dS) = 0.23949			
D8 (n = 82)	—	kappa (ts/tv) = 1.81571	0.25	0	0
		omega (dN/dS) = 0.16717			
D9 (n = 27)	2	kappa (ts/tv) = 1.63182	0.25	0	1
		omega (dN/dS) = 0.23613			
D10 (n = 10)	2	kappa (ts/tv) = 1.84935	0.2	0	2
		omega (dN/dS) = 0.13327			
D11 (n = 34)	—	kappa (ts/tv) = 2.32344	0.3	0	0
		omega (dN/dS) = 0.25915			
M1 (n = 12)	2	kappa (ts/tv) = 1.95556	0.18	0	0
		omega (dN/dS) = 0.04435			
M2 (n = 12)	2	kappa (ts/tv) = 2.26036	0.17	1	1
		omega (dN/dS) = 0.06837			
M3 (n = 7)	1	kappa (ts/tv) = 2.81331	0.29	7	3
		omega (dN/dS) = 0.20874			
M4 (n = 17)	1	kappa (ts/tv) = 2.26629	0.29	0	1
		omega (dN/dS) = 0.06631			
M5 (n = 22)	2	kappa (ts/tv) = 1.66804	0.3	0	2
		omega (dN/dS) = 0.06220			
M6 (n = 21)	2	kappa (ts/tv) = 1.95398	0.18	0	0
		omega (dN/dS) = 0.09371			
M7 (n = 17)	2	kappa (ts/tv) = 1.69771	0.31	4	2
		omega (dN/dS) = 0.11889			
M8 (n = 8)	2	kappa (ts/tv) = 2.24502	0.28	1	1
		omega (dN/dS) = 0.13685			
M9 (n = 6)	4	kappa (ts/tv) = 2.89785	0.26	0	0
		omega (dN/dS) = 0.08899			
M10 (n = 7)	7	kappa (ts/tv) = 3.46399	0.25	0	0
		omega (dN/dS) = 0.18943			
HUS1-D	1	kappa (ts/tv) = 2.00941	0.11	0	0
		omega (dN/dS) = 0.13757			
HUS1-M	0	kappa (ts/tv) = 1.85000	0.13	0	0
		omega (dN/dS) = 0.09842			

n represent sequence numbers within these families;ts/tv means transition/transversion rate; dN/ds means non-synonymous/synonymous rate.

## Discussion

*SWEET* genes are ubiquitous in cellular organisms, from monocellular prokaryotes to higher eukaryotes^1–7^. The dramatic expansion of *SWEET* genes in plant taxa indicates their functional importance in plants^7,9,13,19–21,30^. However, to date, only a few plant species have been investigated^7,8,19–21^, and the *SWEET* family has been considered to be an evolutionarily conserved family^7,8^. The accessibility of more high quality genome sequences provides us with an unprecedented chance to analyze this multi-copy gene family in-depth. As sequencing gaps or errors occurred in almost all sequenced genomes, the prediction of a multi-copy gene family may be underestimated. In the present study, 31 well-annotated or well-assembled genome sequences were carefully selected to minimize the impact of these errors. In addition, considering assembling and sequencing errors, the incomplete of genome sequences or errors in phylogeny reconstruction, we allowed for the gene families in our analysis to be missing in up to half of the dicot or monocot genomes (see the Results). *SWEET* homologs were systemically surveyed in 31 representative species, ranging from unicellular aquatic algae to terrestrial higher plants, thereby demonstrating its functional importance and ancient origin. Only one to four *SWEET* homologs were detected in four aquatic algae and seven to 53 homologs were identified in land plants, indicating a rapid gene expansion of the *SWEET* gene family in higher plants (especially in angiosperms). To confirm our findings, another gene family, the *HUS1* gene family, which is required for homologous recombination repair during meiosis, was also identified in 31 species. This gene family displayed a copy number conservation, evidently different than that of *SWEET* genes (Supplementary Fig. S5).

Family expansion is generally generated by gene duplication, which frequently occurs in plant taxa and has been considered to be a source of neo-functionalization and genetic redundancy^24–27,31^. Estimation of the different duplication models that led to the expansion of *SWEET* genes in vascular plants was also conducted, and included WGD/SD, Tandem, proximal and dispersed duplication^25,31^. Each duplicated model is biased for gene retention. Duplicated genes retained after different duplicated mechanisms often show opposite extremes of the spectrum, particularly in terms of their fates and divergence in expression^26,27^. For example, retained WGD duplicates may play a primary role as a buffer of crucial functions, thereby providing evolutionary stability. Dispersed duplications largely contribute to genetic novelty and adaptation to new environments^26,27^. The distinct duplication patterns observed in this study imply various functional differentiations among different species or taxa. Based on our findings, we inferred that ancestral core *SWEET* genes may be predominantly dispersed duplications. Subsequently, WGD/SD and tandem duplications mainly contributed to the expansion of *SWEET* genes in angiosperms. Further molecular evolutionary rate estimations implied that these WGD/SD and tandem duplicated correlated SWEET gene pairs underwent purifying selection.

Gene duplication and expansion are always followed by functional diversification, and functional diversification may play an important role in providing novel genes for adaptation to new environments^24,25,31^. Here, the expansion of *SWEET* genes, as well as their diverse roles in multiple processes, clearly indicates their functional diversification and evolutionary history. Together, these sugar transporters exhibited evolutionary conservation, as indicated by remarkable similarities in the phylogenetic relationships within the species tree among *SWEET* members in 31 species. However, these *SWEET* genes were diversified into four clades. Among these four clades, only clade II exhibited old, ancient member that were evolutionarily related to algae. To better trace the evolutionary history of *SWEET* genes, these four clades were further divided into 11 D and 10 M subfamilies. Ten of the 21 subfamilies had positive selection sites, indicating that they had important functions under positive selection. For example, M7 had two positive selection sites, and *OsSWEET11* and *OsSWEET15* have been shown to contribute to seed filling and size, and are important in breeding and are involved in domestication^32^.

**Figure 6:**
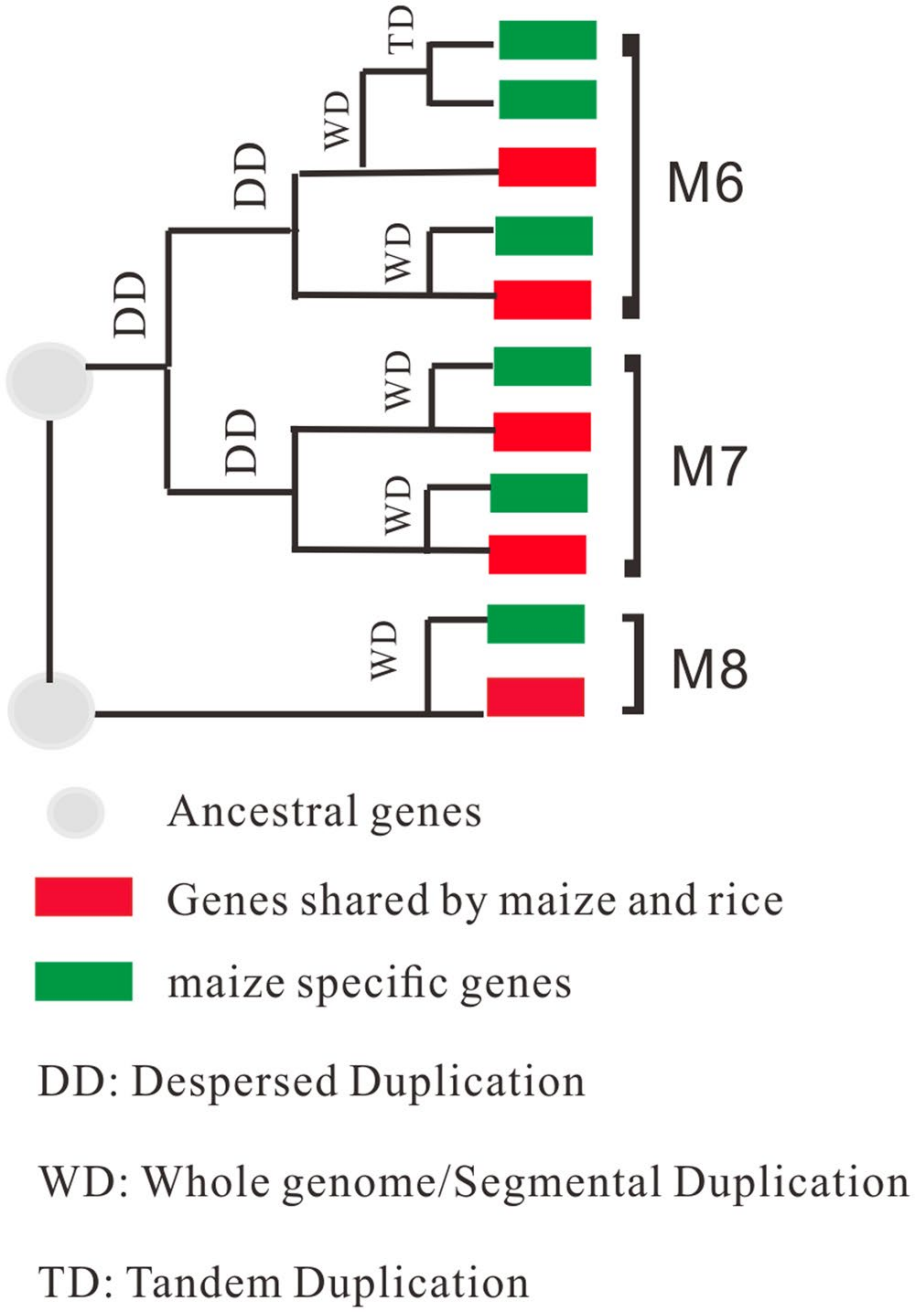
Evolutionary fate of rice and maize SWEET genes in Clade III. According to the phylogenetic tree, no matter monocot or dicot species, two ‘ancestral genes’ were deduced in Clade III. One of these genes was duplicated into two core angiosperm gene pairs (D7, D8 and M6, M7) and the other was retained (D9, M8).

Several *SWEET* genes acting as both transporters and *R*-genes, have attracted the attention of researchers^5,8,17,33,34^. According to our results, clade III harbored three monocot subfamilies, two of which had positive selection sites, indicating positive selection. In clade III, all five rice members were determined to have been targeted by the *Xoo* TAL effectors, thereby inducing pathogenic virulence^18^. Among these, loss-of-function alleles of 3 susceptibility loci (*xa25*, *xa13*, *xa41*) clustered within M7 and M8 have been identified as well-known *R*-genes that are utilized to combat bacterial blight disease^5,17,33^. We can therefore infer that families M7 and M8, or even clade III, may compose a gene pool that can be used for the identification of resistance genes from transporters in various species. Furthermore, arecently evolved hexose transporter gene in wheat (*Triticum aestivum*), *Lr67*, was found to confer partial resistance to three wheat rust pathogen species and powdery mildew; it is a member of the sugar transport proteins (STP) family^34^. Its ortholog in *A*. *thaliana STP13* has also been shown to confer basal resistance to *Botrytis cinerea*^35^. Therefore, the transporters from which pathogens prey on nutrients from the host have been considered to be a genetic reservoir for *R*-genes. Clarifying the evolutionary fate of *SWEET* genes in clade III would be in favor of in-depth function and molecular mechanism analysis of *SWEET* genes. The Subfamilies defined in our study are believed to have been preserved throughout the evolutionary history of angiosperms and are regarded as a reliable core set of *SWEET* genes in angiosperms. No matter monocot or dicot species, two ‘ancestral genes’ were deduced (Fig. 6). One of these genes was duplicated into two core angiosperm gene pairs (D7, D8 and M6, M7) and the other was retained (D9 and M8). Taking the duplication modes that *SWEET* genes are involved in, we aimed to trace the evolutionary fate of *SWEET* genes in clade III, using *SWEET* genes in rice and maize as examples. Clear orthologous relationships were detected between these two species. Interestingly, the five rice homologs were all dispersed duplication correlated genes, while maize has more *SWEET* genes that originated from recently duplication, including WD and TD, and may result in functional redundancy. Different evolutionary fates may result in functional diversity or redundancy. Our results may provide a theoretical basis for further analyses of functional and molecular mechanisms of these *SWEET* genes. Together with our analysis, the engineering of candidate *SWEET* mutants with CRISPR/Cas9 system^36^, could be easily performed during genomic editing of TAL effector target sites, which could be a promising for the exploitation and production of multiple *R*-genes.

## Methods

### Data sources

31 plant genomes and the corresponding gene models and proteomes were downloaded. Herein, annotation resources of *Chlamydomonas reinhardtii*, *Micromonas pusilla*, *Ostreococcus lucimarinus*, *Volvox carteri*, *Physcomitrella patens*, *Sphagnum fallax*, *Selaginella moellendorffii*, *Marchantia polymorpha*, *Musa acuminate*, *Ananas comosus*, *Spirodela polyrhiza*, *Zea mays*, *Sorghum bicolor*, *Brachypodium distachyon*, *Oryza sativa*, *Solanum lycopersicum*, *Medicago truncatula*, *Phaseolus vulgaris*, *Glycine max*, *Prunus persica*, *Malus domestica*, *Populus trichocarpa*, *Eucalyptus grandis*, *Gossypium raimondii*, *Brassica rapa* and *Arabidopsis thaliana* were downloaded from Phytozome (http://phytozome.jgi.doe.gov/pz/portal.html). *Ginkgo biloba* genome was downloaded from Spruce Genome Project database (ftp://plantgenie.org/Data/ConGenIE/Picea_abies/v1.0/). *Amborella trichopoda* genome and its gene models was downloaded from the Amborella Genome Database^23^. *Capsicum annuum* genome was downloaded from the Pepper Genome Database (release 2.0)^37^. *Citrus grandis* and *Citrus sinensis* genome were downloaded from Citrus Genome Database (https://www.citrusgenomedb.org/). Pfam_scan perl script in HMMER3.1 were applied to search all surveyed proteomes against Pfam library^38^. All the hits were first subjected to the Pfam database with an E-value setting of 1.0^39^. *HUS1* genes were identified from 30 surveyed species by the same method to serve as a reference gene family.

### Genome Synteny and Gene duplication

MCScanX, a package developed by the Plant Genome Duplication Database (http://chibba.agtec.uga.edu/duplication/)^28^, was used to evaluate the whole-genome BLASTP results to compute syntenic blocks within or among species. MCScanX can efficiently classify duplicate gene origins within a gene family, including dispersed, proximal, tandem and segmental/WGD duplicates depending on their copy number and genomic distribution. We employed MCScanX to perform synteny analysis and estimate the duplication models in fine-assembled plant genomes (fine-assembled plant genomes means corresponding plant genome sequences had been assembled into pseudomolecule scales).

### Phylogenetic analysis

The ML method was used to build phylogenetic trees using the amino acid sequences of the entire CDS sequences by PhyML 3.0. All the sequences were first aligned using MAFFT with the auto strategy^40^. As there were too many gaps in the alignments of the entire protein sequences, trimAl v1.2 was used to delete gaps with parameter of -automated1^41^ (Additional file 3). Then aligned sequences were further tested to select the best-fit amino acid substitution model for constructing the ML phylogenetic tree by using ProtTest 3.4^42^. The most appropriate model estimated with ProtTest 3.4 was JTT + G + F (−lnL = 44530.08). Finally, trees were constructed with the reliability of internal nodes and evaluated by using Shimodaira-Hasegawa approximate likelihood ratio test (SH-aLRT) values^43^. Other criteria were set according to the results of ProtTest (gamma shape = 1.257; amino acid frequencies = observed). Obtained trees were edited with MEGA 5.0.

To decipher molecular evolutionary genetic basis of *SWEET* genes, their nucleotides of CDS were selected from gene model sequences of all surveyed species by a perl script. Then nucleotides of each CDS were submitted to GUIDANCE2^44^ website and firstly translated to amino acid sequences and aligned by MAFFT. This aligned amino acid sequences were re-transferred to nucleotide sequences. Finally, unreliable alignments were masked by N with a cutoff (0.90). All the following analysis were conducted with these masked alignments. The HyPhy package with the Genetic Algorithm for Recombination Detection (GARD) method as implemented on the Data Monkey webserver (http://www.datamonkey.org/)^45,46^ was used to detect break point sites, which indicated points of unequal crossover.

The codon-based maximum likelihood (CodeML) method in the PAML4.0 package and MEGA 5.0 were firstly used to estimate the average dn/ds ratio of genes within each sub-families^47^. A branch evolutionary analysis for positive selection was conducted using CodeML for average dn/ds of the genes in the M and D sub-families with one-ration model. All masked aligned CDS in each sub-families were used to reconstruct consensus trees for molecular genetic analysis by Seqboot, Dnadist, neighbor and consense program in Phylip package^48^.

To identify the probabilities of sites under positive selection in each sub-families, site models (M7 vs. M8) were implemented in which ω could vary among sites^49^. We used estimated transition/tranversion rates and the F3×4 codon frequencies algorithm as the codon substitution models in the PAML program. Additionally, all of the positively selected sites in the site and branch-site models were identified by using Bayes Empirical Bayes (BEB) analysis with posterior probabilities ≥0.80^47^. Furthermore, positively selected sites were also deduced in the Datamonkey web server by the random effect likelihood (REL) method^45^. Candidate sites under positive selection were defined as those with Bayes factor >50 for REL^45^.

## Electronic supplementary material

Supplemental Information

Supplementary Dataset 1

Supplementary Dataset 2

## Data Availability

All data employed in the present study were downloaded from public databases, which we depicted in methods and materials part of our manuscript. Genomes used for identifying *SWEET* genes were listed in the Supplementary Table S1. Sequence alignments used for phylogenetic tree were provided as Supplementary Dataset 2.

## References

[1] Gamas P, Niebel FDC, Lescure N, Cullimore JV (1996). Use of a subtractive hybridization approach to identify new Medicago truncatula genes induced during root nodule development. Mol Plant Microbe In.

[2] Artero RD (1998). Saliva, a new Drosophila gene expressed in the embryonic salivary glands with homologues in plants and vertebrates. Mechanisms of development.

[3] Dong M (2005). Identification and characterisation of a homolog of an activation gene for the recombination activating gene 1 (RAG 1) in amphioxus. Fish & shellfish immunology.

[4] Hamada M, Wada S, Kobayashi K, Satoh N (2005). Ci-Rga, a gene encoding an MtN3/saliva family transmembrane protein, is essential for tissue differentiation during embryogenesis of the ascidian Ciona intestinalis. Differentiation.

[5] Chu Z (2006). Targeting xa13, a recessive gene for bacterial blight resistance in rice. T*AG*. *Theoretical and applied genetics*. Theoretische und angewandte Genetik.

[6] Guan YF (2008). Ruptured Pollen Grain 1, a member of the MtN3/saliva gene family, is crucial for exine pattern formation and cell integrity of microspores in arabidopsis. Plant Physiol.

[7] Chen LQ (2010). Sugar transporters for intercellular exchange and nutrition of pathogens. Nature.

[8] Yuan M, Wang SP (2013). Rice MtN3/Saliva/SWEET Family Genes and Their Homologs in Cellular Organisms. Mol Plant.

[9] Chen LQ (2012). Sucrose Efflux Mediated by SWEET Proteins as a Key Step for Phloem. Transport. Science.

[10] Fu Q, Cheng L, Guo Y, Turgeon R (2011). Phloem loading strategies and water relations in trees and herbaceous plants. Plant Physiol.

[11] Franceschi VR, Giaquinta RT (1983). Specialized Cellular Arrangements in Legume Leaves in Relation to Assimilate Transport and Compartmentation - Comparison of the Paraveinal Mesophyll. Planta.

[12] Ayre BG (2011). Membrane-transport systems for sucrose in relation to whole-plant carbon partitioning. Mol Plant.

[13] Chen LQ (2014). SWEET sugar transporters for phloem transport and pathogen nutrition. The New phytologist.

[14] Sosso D (2015). Seed filling in domesticated maize and rice depends on SWEET-mediated hexose transport. Nature genetics.

[15] Sutton PN, Henry MJ, Hall JL (1999). Glucose, and not sucrose, is transported from wheat to wheat powdery mildew. Planta.

[16] Yang B, Sugio A, White FF (2006). Os8N3 is a host disease-susceptibility gene for bacterial blight of rice. P Natl Acad Sci USA.

[17] Hutin M, Sabot F, Ghesquière A, Koebnik R, Szurek B (2015). A knowledge-based molecular screen uncovers a broad-spectrum OsSWEET14 resistance allele to bacterial blight from wild rice. The Plant Journal.

[18] Streubel J (2013). Five phylogenetically close rice SWEET genes confer TAL effector-mediated susceptibility to Xanthomonas oryzae pv. oryzae. The New phytologist.

[19] Yuan M (2014). Rice MtN3/saliva/SWEET gene family: Evolution, expression profiling, and sugar transport. Journal of integrative plant biology.

[20] Feng CY, Han JX, Han XX, Jiang J (2015). Genome-wide identification, phylogeny, and expression analysis of the SWEET gene family in tomato. Gene.

[21] Patil, G. *et al*. Soybean (Glycine max) SWEET gene family: insights through comparative genomics, transcriptome profiling and whole genome re-sequence analysis. *Bmc Genomics* **16**, 10.1186/S12864-015-1730-Y (2015). 10.1186/s12864-015-1730-y PMC4499210 26162601

[22] Rensing SA (2008). The Physcomitrella genome reveals evolutionary insights into the conquest of land by plants. Science.

[23] Amborella Genome P (2013). The Amborella genome and the evolution of flowering plants. Science.

[24] Freeling M (2009). Bias in plant gene content following different sorts of duplication: tandem, whole-genome, segmental, or by transposition. Annual review of plant biology.

[25] Innan H, Kondrashov F (2010). The evolution of gene duplications: classifying and distinguishing between models. Nat Rev Genet.

[26] Wang, Y. P. *et al*. Modes of Gene Duplication Contribute Differently to Genetic Novelty and Redundancy, but Show Parallels across Divergent Angiosperms. *Plos One* **6**, 10.1371/journal.pone.0028150 (2011). 10.1371/journal.pone.0028150 PMC3229532 22164235

[27] Wang Y, Wang X, Paterson AH (2012). Genome and gene duplications and gene expression divergence: a view from plants. Annals of the New York Academy of Sciences.

[28] Wang Y (2012). MCScanX: a toolkit for detection and evolutionary analysis of gene synteny and collinearity. Nucleic acids research.

[29] Tao Y (2015). Structure of a eukaryotic SWEET transporter in a homotrimeric complex. Nature.

[30] Xuan YH (2013). Functional role of oligomerization for bacterial and plant SWEET sugar transporter family. Proc Natl Acad Sci USA.

[31] Bowers JE, Chapman BA, Rong J, Paterson AH (2003). Unravelling angiosperm genome evolution by phylogenetic analysis of chromosomal duplication events. Nature.

[32] Yang JL, Luo DP, Yang B, Frommer WB, Eom JS (2018). SWEET11 and 15 as key players in seed filling in rice. New Phytologist.

[33] Liu QS (2011). A paralog of the MtN3/saliva family recessively confers race-specific resistance to Xanthomonas oryzae in rice. Plant Cell Environ..

[34] Moore, J. W. *et al*. A recently evolved hexose transporter variant confers resistance to multiple pathogens in wheat. *Nature genetics*, 10.1038/ng.3439 (2015). 10.1038/ng.3439 26551671

[35] Lemonnier P (2014). Expression of Arabidopsis sugar transport protein STP13 differentially affects glucose transport activity and basal resistance to Botrytis cinerea. Plant molecular biology.

[36] Shalem O, Sanjana NE, Zhang F (2015). High-throughput functional genomics using CRISPR-Cas9. Nat Rev Genet.

[37] Qin C (2014). Whole-genome sequencing of cultivated and wild peppers provides insights into Capsicum domestication and specialization. P Natl Acad Sci USA.

[38] Eddy SR (2011). Accelerated Profile HMM Searches. PLoS computational biology.

[39] Finn RD (2014). Pfam: the protein families database. Nucleic acids research.

[40] Katoh K, Misawa K, Kuma K, Miyata T (2002). MAFFT: a novel method for rapid multiple sequence alignment based on fast Fourier transform. Nucleic acids research.

[41] Capella-Gutierrez S, Silla-Martinez JM, Gabaldon T (2009). trimAl: a tool for automated alignment trimming in large-scale phylogenetic analyses. Bioinformatics.

[42] Darriba D, Taboada GL, Doallo R, Posada D (2011). ProtTest 3: fast selection of best-fit models of protein evolution. Bioinformatics.

[43] Guindon S (2010). New algorithms and methods to estimate maximum-likelihood phylogenies: assessing the performance of PhyML 3.0. Systematic biology.

[44] Sela I, Ashkenazy H, Katoh K, Pupko T (2015). Guidance 2: accurate detection of unreliable alignment regions accounting for the uncertainty of multiple parameters. Nucleic acids research.

[45] Pond SLK, Frost SDW (2005). Datamonkey: rapid detection of selective pressure on individual sites of codon alignments. Bioinformatics.

[46] Delport W, Poon AFY, Frost SDW, Pond SLK (2010). Datamonkey 2010: a suite of phylogenetic analysis tools for evolutionary biology. Bioinformatics.

[47] Yang Z (2007). PAML 4: phylogenetic analysis by maximum likelihood. Mol Biol Evol.

[48] Felsenstein J (1981). Evolutionary trees from DNA sequences: a maximum likelihood approach. J Mol Evol.

[49] Yang Z, Nielsen R (2000). Estimating synonymous and nonsynonymous substitution rates under realistic evolutionary models. Mol Biol Evol.

