# Supplementary material for: Deciphering evolutionary dynamics of SWEET genes in diverse plant lineages
Source: Sci Rep. 2018 Sep 7;8:13440. doi: 10.1038/s41598-018-31589-x (PMC6128921; doi:10.1038/s41598-018-31589-x)

Supplementary Information

Deciphering evolutionary dynamics of *SWEET* genes in diverse plant lineages

Xiaoyu Li[1,2] Weina Si[1,2] QianQianQin[1] HaoWu[1] Haiyang Jiang[1,*]

[1]Key Laboratory of Crop Biology of Anhui Province, Anhui Agricultural University, Hefei 230036, China; [2]These authors contributed equally to this work

Supplementary Figure S1: Relationship between SWEET gene number and (a) Gene loci numbers on the whole genome scale, (b) Genome size, (c) Chromosome numbers, respectively.

Supplementary Figure S2: The distribution and inter-genomic comparison of SWEET genes in two legume species.

Supplementary Figure S3: Diagram of SWEET proteins harboring one(a), two(b) and three MtN3_slv(c) domains.

Supplementary Figure S4: The maximum-likelihood (ML) phylogenetic tree built by the *SWEET* genes with entire protein sequences from 31 plant species.

Supplementary Figure S5: The maximum-likelihood (ML) phylogenetic tree built by the *HUS1* genes with entire protein sequences from 31 plant species.

Supplementary Table S1: Genomic information of surveyed 30 species.

Supplementary Figure S1: Relationship between SWEET gene number and (a) Gene loci numbers on the whole genome scale, (b) Genome size, (c) Chromosome numbers, respectively.

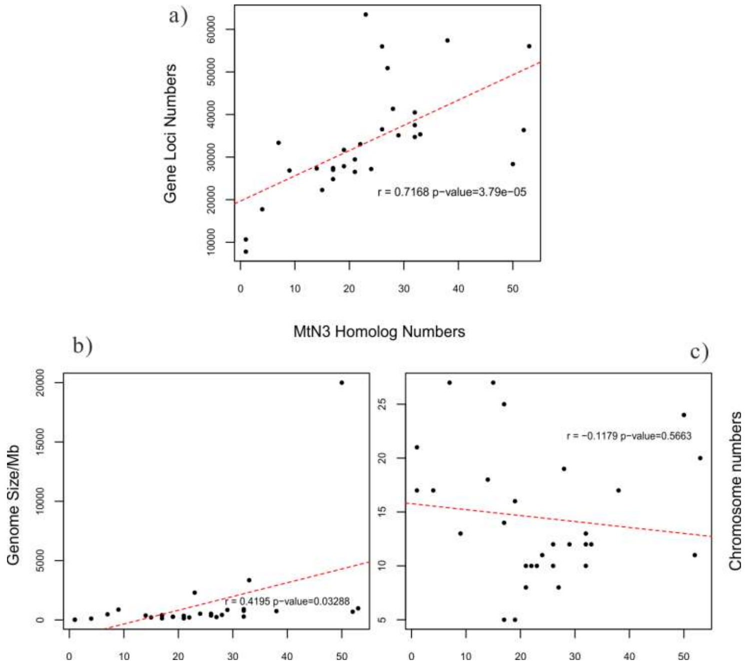

Supplementary S2 The distribution and inter-genomic comparison of SWEET genes in two legume species.

Gene synteny in *G.max* (Gm) (a) and *M. truncatula* (Mt) (b). The gray lines represent whole genome duplication (WGD) blocks within each species. *SWEET* homologs marked with blue, red, green, purple, represent that they were resulted from dispersed, tandem, WGD/segmental, and other types duplication, respectively.

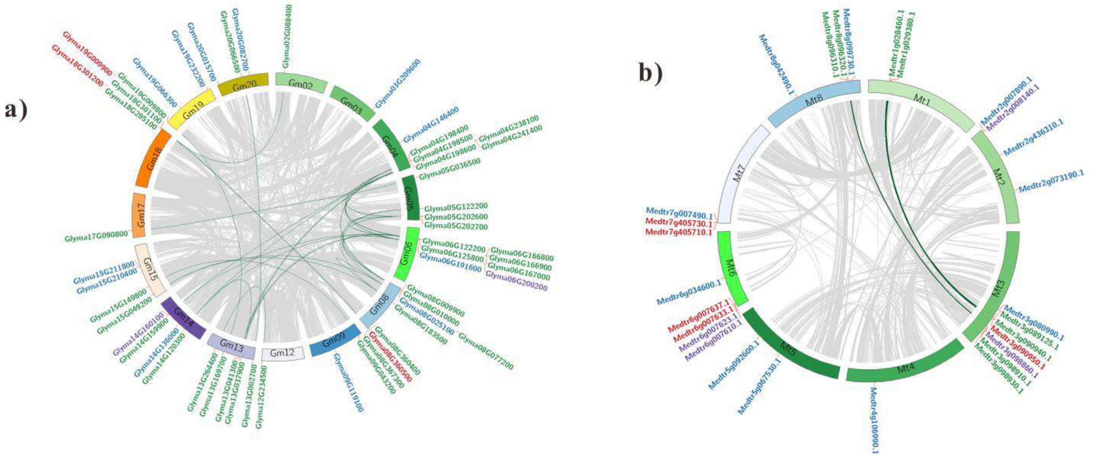

Supplementary Figure S3: Diagram of SWEET proteins harboring one(a), two(b) and three MtN3_slv(c) domains.

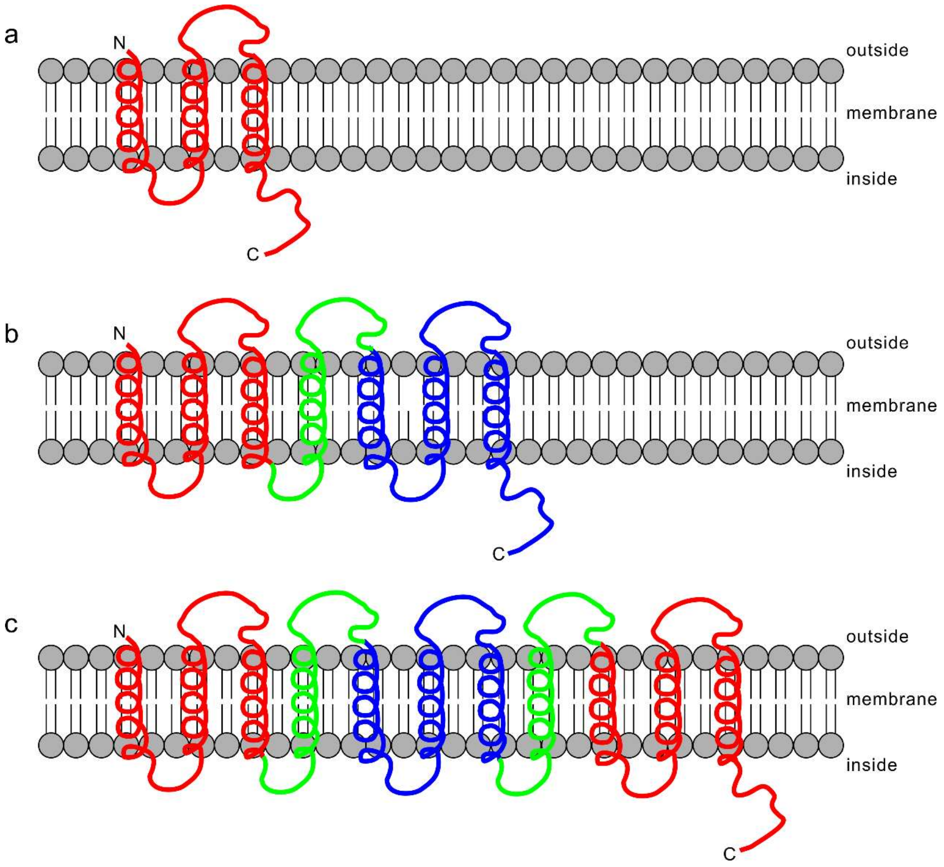

Supplementary Figure S4: The maximum-likelihood (ML) phylogenetic tree built by the *SWEET* genes with entire protein sequences from 31 plant species. Genes from different species have corresponding prefix, which were listed in Table S1. Branch with different color represents different plant species. Green indicates dicot species; azure indicates monocot species; black represents *A.trichopoda* and *A.coerulea*; red indicates *P.abies*; rosy red indicates *S.moellendorffii*; brown represents *P.patens*; yellow represents algae species. Classification of four clades were also marked by square brackets.

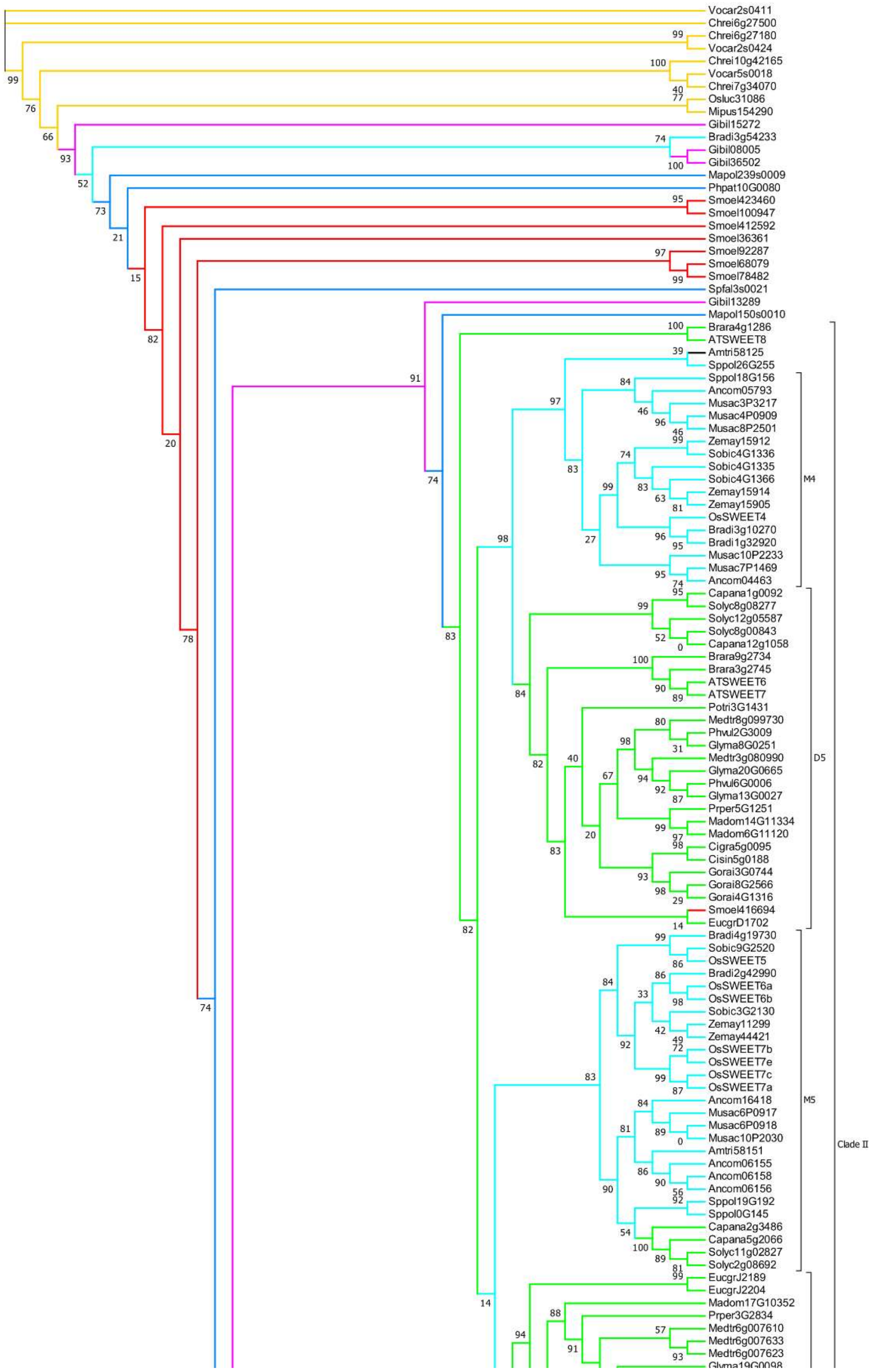

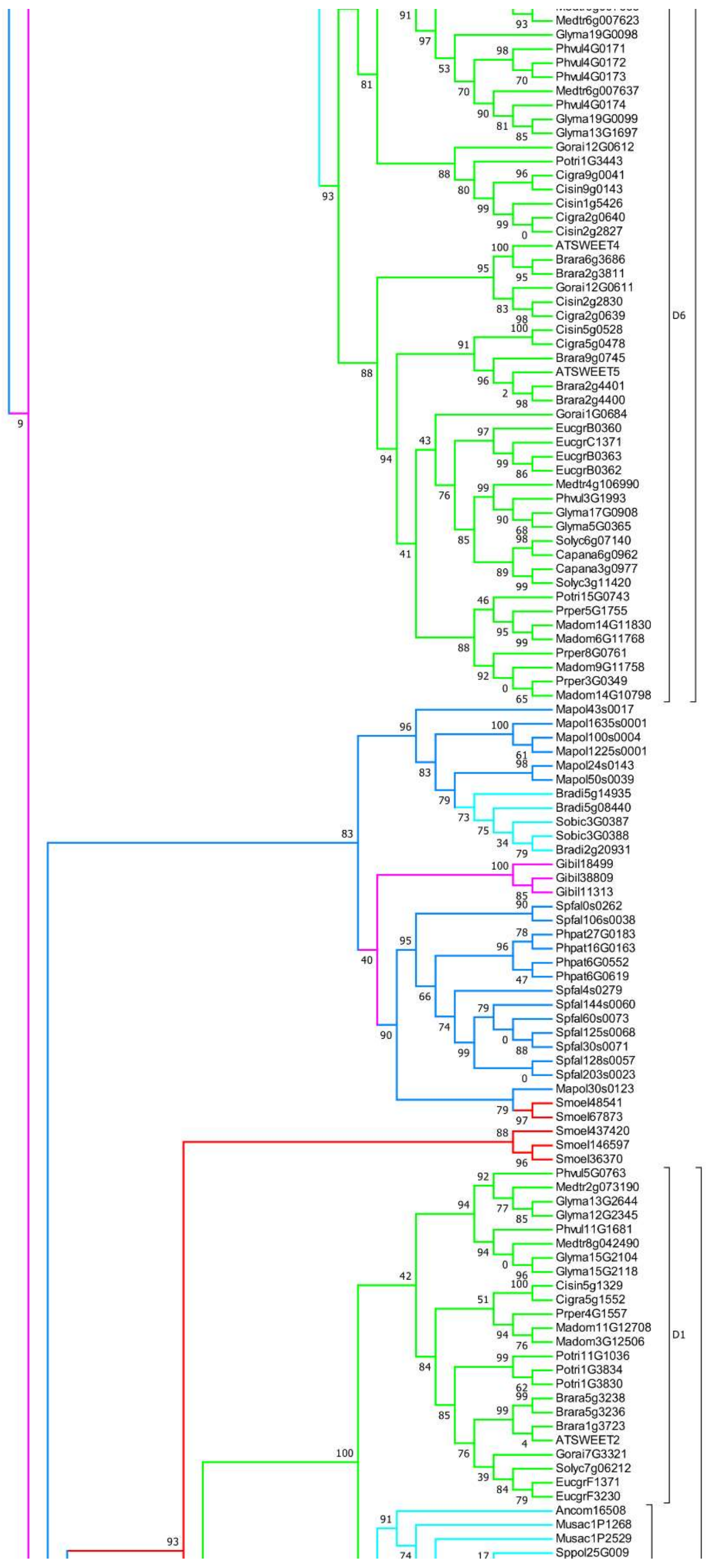

Medtr6g007623
Glyma19G0098
Phvul4G0171
Phvul4G0172
Phvul4G0173
Medtr6g007637
Phvul4G0174
Glyma19G0099
Glyma13G1697
Gorai12G0612
Potri1G3443
Cigra9g0041
Cisin9g0143
Cisin1g5426
Cigra2g0640
Cisin2g2827
ATSWEET4
Brara6g3686
Brara2g3811
Gorai12G0611
Cisin2g2830
Cigra2g0639
Cisin5g0528
Cigra5g0478
Brara9g0745
ATSWEET5
Brara2g4401
Brara2g4400
Gorai1G0684
EucgrB0360
EucgrC1371
EucgrB0363
EucgrB0362
Medtr4g106990
Phvul3G1993
Glyma17G0908
Glyma5G0365
Solyc6g07140
Capana6g0962
Capana3g0977
Solyc3g11420
Potri15G0743
Prper5G1755
Madom14G11830
Madom6G11768
Prper8G0761
Madom9G11758
Prper3G0349
Madom14G10798
D6
Mapol43s0017
Mapol1635s0001
Mapol100s0004
Mapol1225s0001
Mapol24s0143
Mapol50s0039
Bradi5g14935
Bradi5g08440
Sobic3G0387
Sobic3G0388
Bradi2g20931
Gibil18499
Gibil38809
Gibil11313
Spfal0s0262
Spfal106s0038
Phpat27G0183
Phpat16G0163
Phpat6G0552
Phpat6G0619
Spfal4s0279
Spfal144s0060
Spfal60s0073
Spfal125s0068
Spfal30s0071
Spfal128s0057
Spfal203s0023
Mapol30s0123
Smoel48541
Smoel67873
Smoel437420
Smoel146597
Smoel36370
Phvul5G0763
Medtr2g073190
Glyma13G2644
Glyma12G2345
Phvul11G1681
Medtr8g042490
Glyma15G2104
Glyma15G2118
Cisin5g1329
Cigra5g1552
Prper4G1557
Madom11G12708
Madom3G12506
Potri11G1036
Potri1G3834
Potri1G3830
Brara5g3238
Brara5g3236
Brara1g3723
ATSWEET2
Gorai7G3321
Solyc7g06212
EucgrF1371
EucgrF3230
D1
Ancom16508
Musac1P1268
Musac1P2529
Sppol25G009

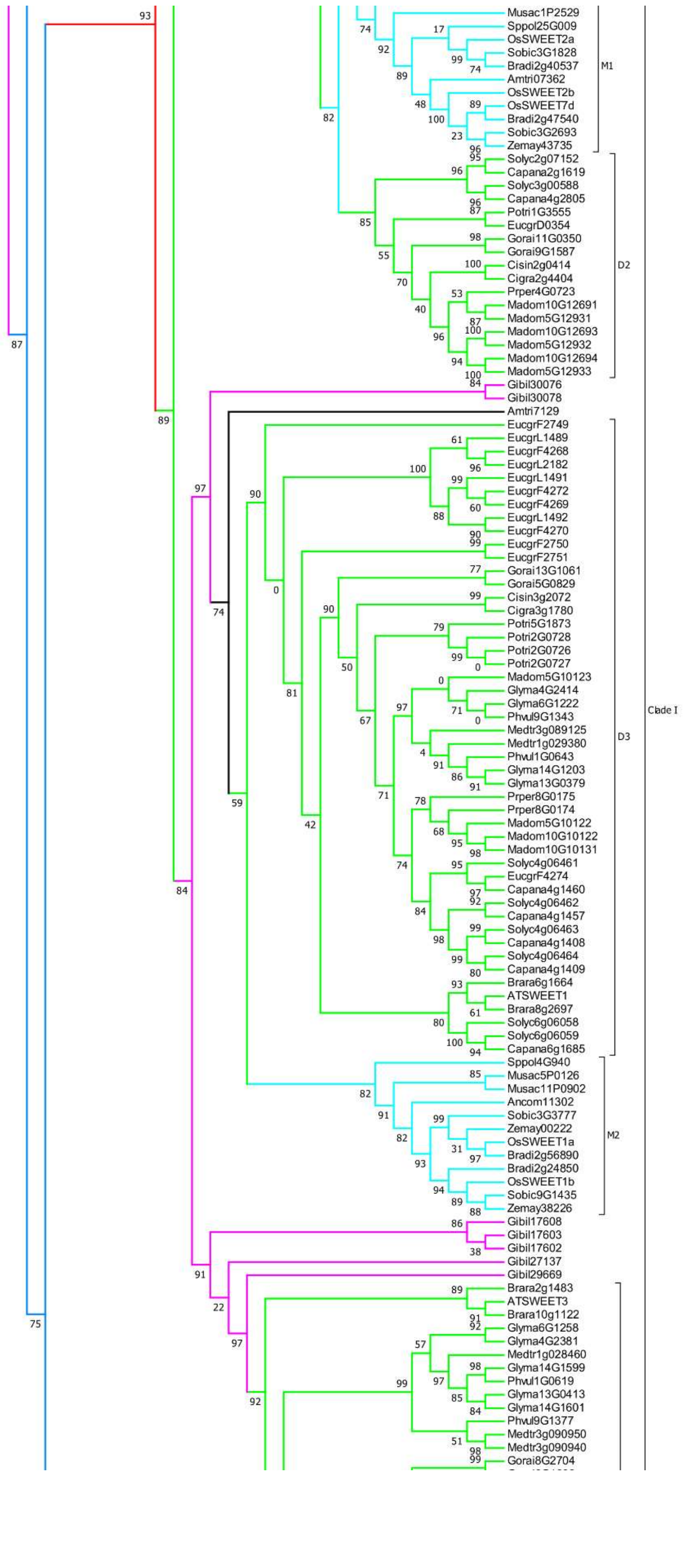
Musac1P2529
Sppol25G009
OsSWEET2a
Sobic3G1828
Bradi2g40537
Amtri07362
OsSWEET2b
OsSWEET7d
Bradi2g47540
Sobic3G2693
Zemay43735
M1
Solyc2g07152
Capana2g1619
Solyc3g00588
Capana4g2805
Potri1G3555
EucgrD0354
Gorai11G0350
Gorai9G1587
Cisin2g0414
Cigra2g4404
Prper4G0723
Madom10G12691
Madom5G12931
Madom10G12693
Madom5G12932
Madom10G12694
Madom5G12933
D2
Gibil30076
Gibil30078
Amtri7129
EucgrF2749
EucgrL1489
EucgrF4268
EucgrL2182
EucgrL1491
EucgrF4272
EucgrF4269
EucgrL1492
EucgrF4270
EucgrF2750
EucgrF2751
Gorai13G1061
Gorai5G0829
Cisin3g2072
Cigra3g1780
Potri5G1873
Potri2G0728
Potri2G0726
Potri2G0727
Madom5G10123
Glyma4G2414
Glyma6G1222
Phvul9G1343
Medtr3g089125
Medtr1g029380
Phvul1G0643
Glyma14G1203
Glyma13G0379
Prper8G0175
Prper8G0174
Madom5G10122
Madom10G10122
Madom10G10131
Solyc4g06461
EucgrF4274
Capana4g1460
Solyc4g06462
Capana4g1457
Solyc4g06463
Capana4g1408
Solyc4g06464
Capana4g1409
Brara6g1664
ATSWEET1
Brara8g2697
Solyc6g06058
Solyc6g06059
Capana6g1685
D3
Clade I
Sppol4G940
Musac5P0126
Musac11P0902
Ancom11302
Sobic3G3777
Zemay00222
OsSWEET1a
Bradi2g56890
Bradi2g24850
OsSWEET1b
Sobic9G1435
Zemay38226
M2
Gibil17608
Gibil17603
Gibil17602
Gibil27137
Gibil29669
Brara2g1483
ATSWEET3
Brara10g1122
Glyma6G1258
Glyma4G2381
Medtr1g028460
Glyma14G1599
Phvul1G0619
Glyma13G0413
Glyma14G1601
Phvul9G1377
Medtr3g090950
Medtr3g090940
Gorai8G2704

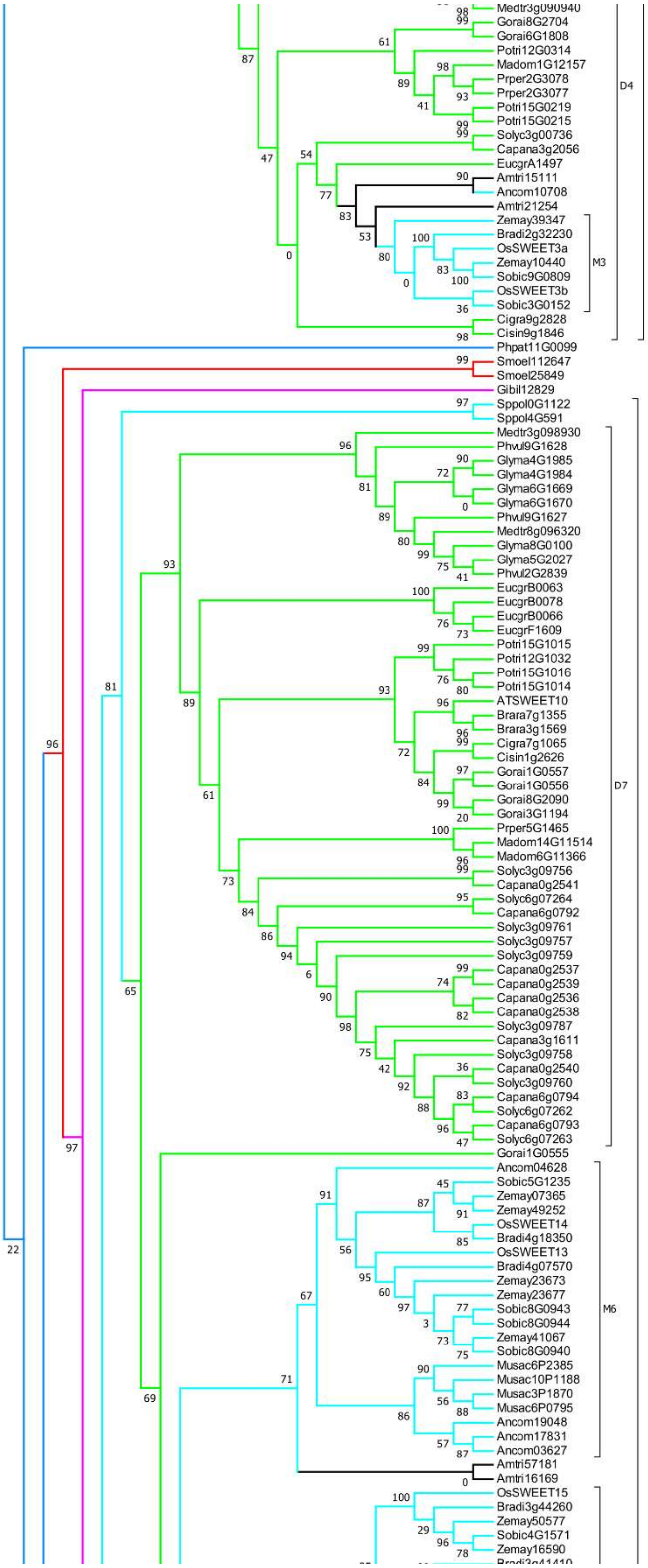
Medtr3g090940
Gorai8G2704
Gorai6G1808
Potri12G0314
Madom1G12157
Prper2G3078
Prper2G3077
Potri15G0219
Potri15G0215
Solyc3g00736
Capana3g2056
EucgrA1497
Amtri15111
Ancom10708
Amtri21254
Zemay39347
Bradi2g32230
OsSWEET3a
Zemay10440
Sobic9G0809
OsSWEET3b
Sobic3G0152
Cigra9g2828
Cisin9g1846
D4
M3
Phpat11G0099
Smoel112647
Smoel25849
Gibil12829
Sppol0G1122
Sppol4G591
Medtr3g098930
Phvul9G1628
Glyma4G1985
Glyma4G1984
Glyma6G1669
Glyma6G1670
Phvul9G1627
Medtr8g096320
Glyma8G0100
Glyma5G2027
Phvul2G2839
EucgrB0063
EucgrB0078
EucgrB0066
EucgrF1609
Potri15G1015
Potri12G1032
Potri15G1016
Potri15G1014
ATSWEET10
Brara7g1355
Brara3g1569
Cigra7g1065
Cisin1g2626
Gorai1G0557
Gorai1G0556
Gorai8G2090
Gorai3G1194
Prper5G1465
Madom14G11514
Madom6G11366
Solyc3g09756
Capana0g2541
Solyc6g07264
Capana6g0792
Solyc3g09761
Solyc3g09757
Solyc3g09759
Capana0g2537
Capana0g2539
Capana0g2536
Capana0g2538
Solyc3g09787
Capana3g1611
Solyc3g09758
Capana0g2540
Solyc3g09760
Capana6g0794
Solyc6g07262
Capana6g0793
Solyc6g07263
D7
Gorai1G0555
Ancom04628
Sobic5G1235
Zemay07365
Zemay49252
OsSWEET14
Bradi4g18350
OsSWEET13
Bradi4g07570
Zemay23673
Zemay23677
Sobic8G0943
Sobic8G0944
Zemay41067
Sobic8G0940
Musac6P2385
Musac10P1188
Musac3P1870
Musac6P0795
Ancom19048
Ancom17831
Ancom03627
M6
Amtri57181
Amtri16169
OsSWEET15
Bradi3g44260
Zemay50577
Sobic4G1571
Zemay16590

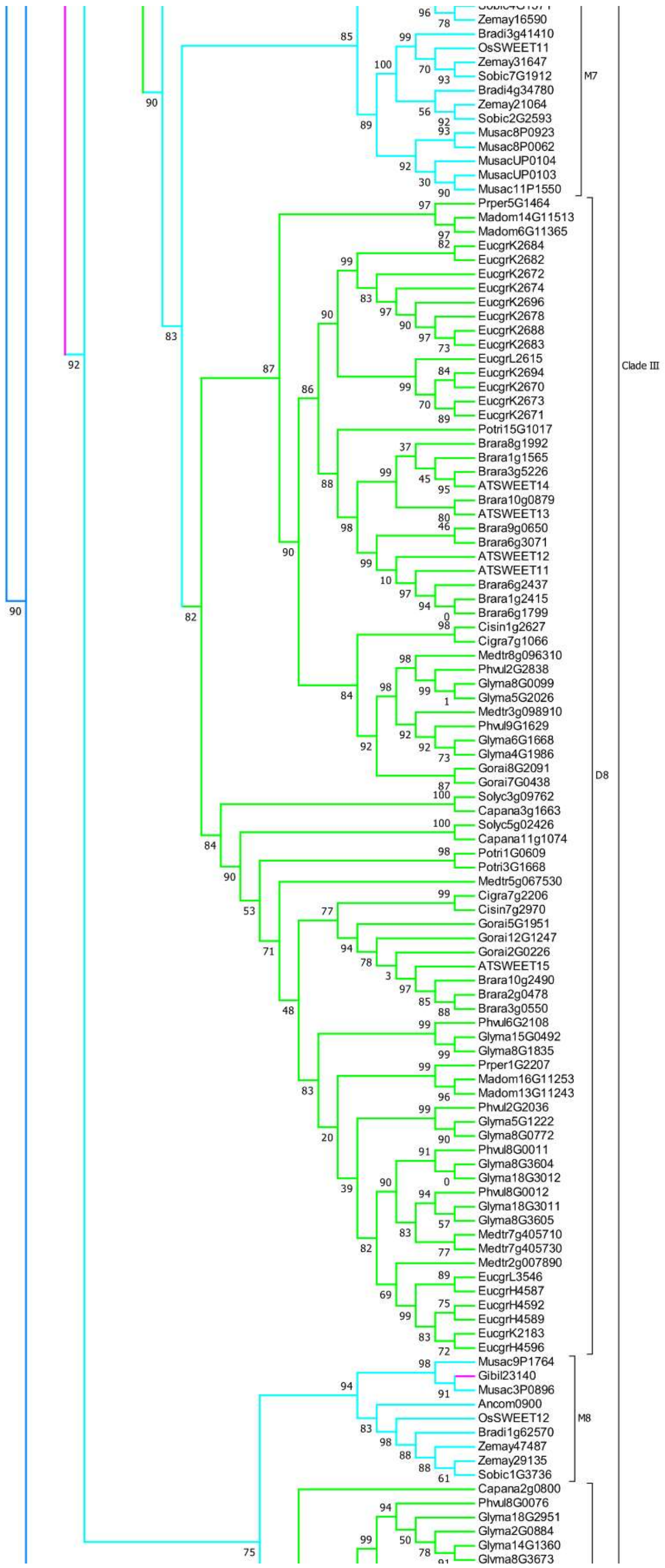

Zemay16590
Bradi3g41410
OsSWEET11
Zemay31647
Sobic7G1912
Bradi4g34780
Zemay21064
Sobic2G2593
Musac8P0923
Musac8P0062
MusacUP0104
MusacUP0103
Musac11P1550
M7
Prper5G1464
Madom14G11513
Madom6G11365
EucgrK2684
EucgrK2682
EucgrK2672
EucgrK2674
EucgrK2696
EucgrK2678
EucgrK2688
EucgrK2683
EucgrL2615
EucgrK2694
EucgrK2670
EucgrK2673
EucgrK2671
Potri15G1017
Brara8g1992
Brara1g1565
Brara3g5226
ATSWEET14
Brara10g0879
ATSWEET13
Brara9g0650
Brara6g3071
ATSWEET12
ATSWEET11
Brara6g2437
Brara1g2415
Brara6g1799
Cisin1g2627
Cigra7g1066
Medtr8g096310
Phvul2G2838
Glyma8G0099
Glyma5G2026
Medtr3g098910
Phvul9G1629
Glyma6G1668
Glyma4G1986
Gorai8G2091
Gorai7G0438
Solyc3g09762
Capana3g1663
Solyc5g02426
Capana11g1074
Potri1G0609
Potri3G1668
Medtr5g067530
Cigra7g2206
Cisin7g2970
Gorai5G1951
Gorai12G1247
Gorai2G0226
ATSWEET15
Brara10g2490
Brara2g0478
Brara3g0550
Phvul6G2108
Glyma15G0492
Glyma8G1835
Prper1G2207
Madom16G11253
Madom13G11243
Phvul2G2036
Glyma5G1222
Glyma8G0772
Phvul8G0011
Glyma8G3604
Glyma18G3012
Phvul8G0012
Glyma18G3011
Glyma8G3605
Medtr7g405710
Medtr7g405730
Medtr2g007890
EucgrL3546
EucgrH4587
EucgrH4592
EucgrH4589
EucgrK2183
EucgrH4596
D8
Clade III
Musac9P1764
Gibil23140
Musac3P0896
Ancom0900
OsSWEET12
Bradi1g62570
Zemay47487
Zemay29135
Sobic1G3736
M8
Capana2g0800
Phvul8G0076
Glyma18G2951
Glyma2G0884
Glyma14G1360
Glyma8G3673

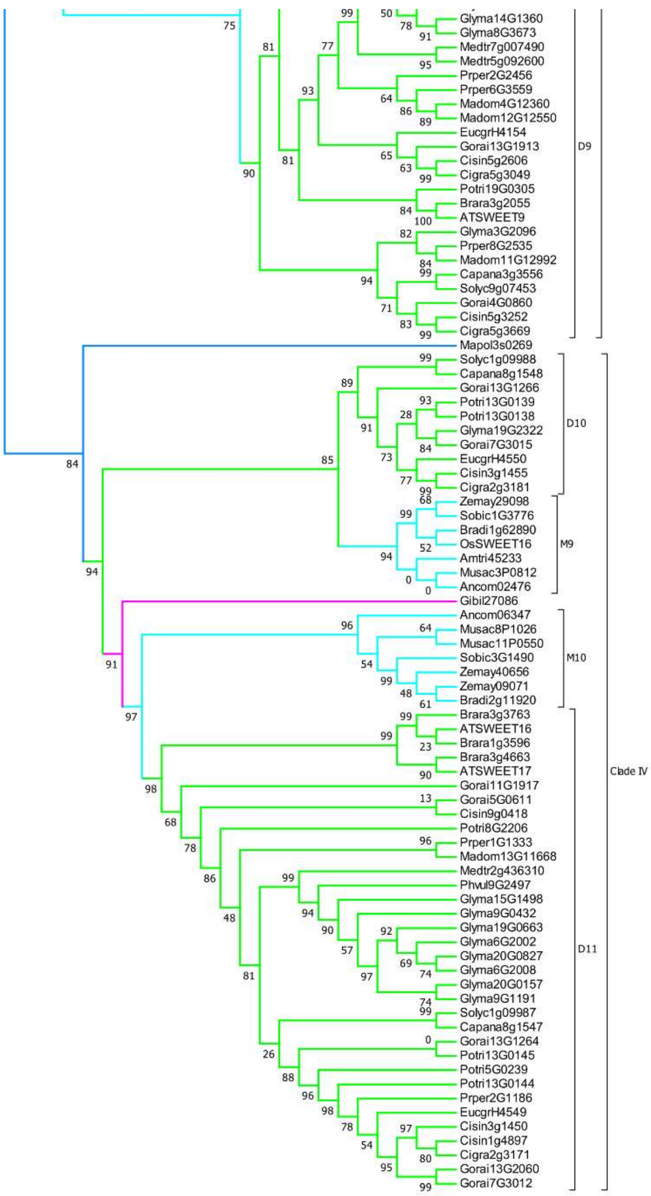

Glyma14G1360
Glyma8G3673
Medtr7g007490
Medtr5g092600
Prper2G2456
Prper6G3559
Madom4G12360
Madom12G12550
EucgrH4154
Gorai13G1913
Cisin5g2606
Cigra5g3049
Potri19G0305
Brara3g2055
ATSWEET9
Glyma3G2096
Prper8G2535
Madom11G12992
Capana3g3556
Solyc9g07453
Gorai4G0860
Cisin5g3252
Cigra5g3669
D9
Mapol3s0269
Solyc1g09988
Capana8g1548
Gorai13G1266
Potri13G0139
Potri13G0138
Glyma19G2322
Gorai7G3015
EucgrH4550
Cisin3g1455
Cigra2g3181
D10
Zemay29098
Sobic1G3776
Bradi1g62890
OsSWEET16
Amtri45233
Musac3P0812
Ancom02476
M9
Gibil27086
Ancom06347
Musac8P1026
Musac11P0550
Sobic3G1490
Zemay40656
Zemay09071
Bradi2g11920
M10
Brara3g3763
ATSWEET16
Brara1g3596
Brara3g4663
ATSWEET17
Gorai11G1917
Gorai5G0611
Cisin9g0418
Potri8G2206
Prper1G1333
Madom13G11668
Medtr2g436310
Phvul9G2497
Glyma15G1498
Glyma9G0432
Glyma19G0663
Glyma6G2002
Glyma20G0827
Glyma6G2008
Glyma20G0157
Glyma9G1191
Solyc1g09987
Capana8g1547
Gorai13G1264
Potri13G0145
Potri5G0239
Potri13G0144
Prper2G1186
EucgrH4549
Cisin3g1450
Cisin1g4897
Cigra2g3171
Gorai13G2060
Gorai7G3012
D11
Clade IV

Supplementary Figure S5: The maximum-likelihood (ML) phylogenetic tree built by the *HUS1* genes with entire protein sequences from 31 plant species. This tree was also built by PhyML 3.0.

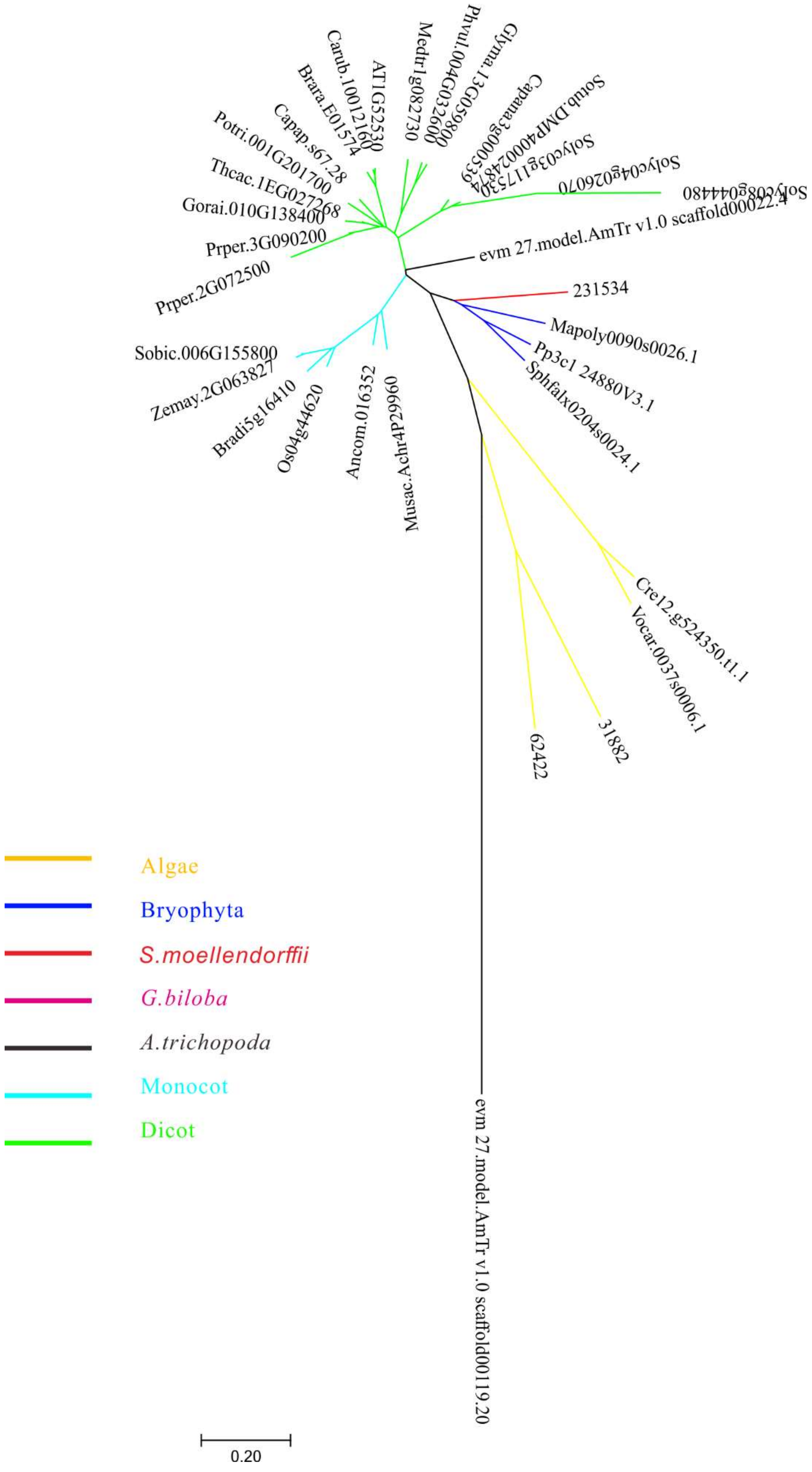

Supplement: Supplementary file 1 — Supplemental Information [file 41598_2018_31589_MOESM1_ESM.pdf]
